# Supplementary material for: Effect of Limosilactobacillus reuteri LRE02–Lacticaseibacillus rhamnosus LR04 Combination on Antibiotic-Associated Diarrhea in a Pediatric Population: A National Survey
Source: J Clin Med. 2020 Sep 24;9(10):3080. doi: 10.3390/jcm9103080 (PMC7650601; doi:10.3390/jcm9103080)
Supplement: Supplementary file 1 [file jcm-09-03080-s001.docx]

Supplemental Table S1. Weight loss stratification. Data are expressed in grams as mean ±SD

| Age | Group A | Group B | *p-value* |
| --- | --- | --- | --- |
| 0-2 years | 330 (±350) | 278 (±226) | 0.129 |
| 2-5 years | 290 (±309) | 307 (±243) | 0.582 |
| 5-10 years | 360 (±186) | 391 (±224) | 0.552 |
| >10 years | 323 (±142) | 362 (±163) | 0.557 |

Supplemental Table S2. Prevalence of AAD in Group A and Group B, respect to antibiotic prescription.

Proportion of patients with AAD in Group A and Group B, according to antibiotic prescription. Chi-square was used for comparison between groups.

|  | **Group A, N (%)** | **Group B, N (%)** | **p value** |
| --- | --- | --- | --- |
| Penicillins | 658 (30.8%) | 1224 (59.7%) | <0.001 |
| Cephalosporins | 357 (20.1%) | 664 (38.9%) | <0.001 |
| Macrolides | 83 (22.3%) | 250 (58.7%) | <0.001 |

Supplemental Table S3. Statistics for figure 5

The stratification among antibiotics for the specific symptom “beginning of diarrhoeic episodes (days)” was not possible for macrolides due to low number of patients (N=1).

| Figure 5A | | | |
| --- | --- | --- | --- |
| Duration of diarrhoeic episodes (days) | Group A | Group B | p-value |
| Penicillins | 3 (IQR 2-3) | 3 (2-4) | <0.001 |
| Cephalosporins | 2 (IQR 2-3) | 3 (2-4) | <0.001 |
| Macrolides | 3 (IQR 2-3) | 3 (2-4) | 0.017 |
| Figure 5B | | | |
| Frequency of evacuation (n/days) |  |  |  |
| Penicillins | 3 (IQR 3-4) | 3 (IQR 3-4) | 0.912 |
| Cephalosporins | 3 (IQR 3-4) | 3 (IQR 3-4) | 0.743 |
| Macrolides | 3 (IQR 3-3) | 3 (IQR 3-4) | 0.035 |
| Figure 5C | | | |
| Beginning of diarrhoeic episodes (days) | Group A | Group B | p-value |
| Penicillins | 2 (IQR 1-2) | 2 (IQR 1-3) | 0.010 |
| Cephalosporins | 1.5 (IQR 1-2) | 4 (IQR 3-5) | 0.013 |
